# Healthcare Workers’ Hand Microbiome May Mediate Carriage of Hospital Pathogens

**DOI:** 10.3390/pathogens3010001

**Published:** 2013-12-27

**Authors:** Mariana Rosenthal, Allison Aiello, Elaine Larson, Carol Chenoweth, Betsy Foxman

**Affiliations:** 1 Department of Epidemiology, School of Public Health, University of Michigan, 1415 Washington Heights, Ann Arbor, MI 48109, USA; E-Mails: rosenbr@umich.edu (M.R.); aielloa@umich.edu (A.A.); cchenow@umich.edu (C.C.); 2 School of Nursing and Department of Epidemiology, Mailman School of Public Health, Columbia University, New York, NY 10032, USA; E-Mail: ell23@columbia.edu

**Keywords:** skin, hand, microbiome, microbiota, hand hygiene, healthcare, pathogen

## Abstract

One function of skin microbiota is to resist colonization and infection by external microorganisms. We sought to detect whether the structure of the hand microbiota of 34 healthcare workers (HCW) in a surgical intensive care unit mediates or modifies the relationship between demographic and behavioral factors and potential pathogen carriage on hands after accounting for pathogen exposure. We used a taxonomic screen (16S rRNA) to characterize the bacterial community, and qPCR to detect presence of *Staphylococcus aureus,* *Enterococcus* spp., methicillin-resistant *Staphylococcus aureus* (MRSA), and *Candida albicans* on their dominant hands. Hands were sampled weekly over a 3-week period. Age, hand hygiene, and work shift were significantly associated with potential pathogen carriage and the associations were pathogen dependent. Additionally, the overall hand microbiota structure was associated with the carriage of potential pathogens. Hand microbiota community structure may act as a biomarker of pathogen carriage, and modifying that structure may potentially limit pathogen carriage among HCW.

## 1. Introduction

Healthcare workers (HCW) are continually exposed to pathogens in their daily work, but for the most part they do not become ill. This is not because HCWs are hardier, but because hand microbiota is intrinsically resistant to colonization and infection by external microorganisms. This intrinsic resistance is modified by a number of factors including hand hygiene, the extent of their exposure to pathogens, and inherent immunocompetency associated with the ecological relationships between the pathogen(s) and the host microbiota.

**Figure 1:**
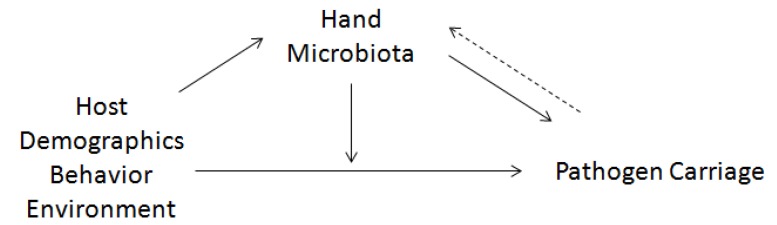
Conceptual framework describing the relationship between potential risk factors for pathogen carriage, pathogen carriage and microbiota.

Human microbiota are associated with host health and disease [1], but most of the evidence supporting this association comes from studies of the gut [2,3]. For microbiota in other systems, including the skin, it is unclear the extent that the microbiota influence one’s capacity to carry or resist a pathogen. To further examine this issue, we evaluated behavioral and environmental risk factors for nosocomial pathogen carriage among 34 HCW in a surgical intensive care unit (SICU). HCWs were sampled weekly over a 3-week period. We assessed whether the carriage of *Staphylococcus aureus*, *Enterococcus* spp., MRSA, and *Candida albicans* was associated with HCW hand microbiota community structure after taking into account known host, behavioral, and environmental risk factors (Figure 1).

## 2. Results and Discussion

### 2.1. Survey Results

The 34 HCW were predominantly female (76.5%), Caucasian (91.1%) and born in the United States (91.1%). They averaged 34.5 years of age (range 20–59), and 7 (20.6%) had at least one child <5 years old living within their household. Twenty-four (70.6%) HCW were Registered Nurses (RN), 6 (17.6%) were Respiratory Specialists, and 4 (11.8%) were Nurse Technologists. 

During a typical 12-h work shift, about half (52.9%) of the HCW reported washing their hands with soap and water 6-20 times, and 41.2% used alcohol rub >40 times. Almost two-thirds (61.8%) reported donning >40 pairs of gloves during a typical 12-hr work shift, mostly nitrile, powder-free. Almost two-thirds (64.7%) reported using lotion or moisturizer on their hands 1–5 times per 12-hr work shift. 

Most of the HCW (97.1%) rated themselves to be in excellent or good overall health. Their hand health was also good: using the Visual Scoring of Skin Scale the majority had minimal or no scaling with only 6 (17.7%) with slightly scaly hands. This is consistent with self-reports using the Hand Skin Assessment Scale, where most rated their hands at least a 6 (out of 7) on appearance (55.9%), intactness (38.2%), and moisture content (32.4%), and a 7 on sensation (64.7%).

Over half (58.8%) cared directly for an average of 1–2 patients per 12-h work shift. Many took vital signs (73.5%) and turned patients (55.9%) >10 times per 12-h work shift. Tasks performed by HCW 1–4 times per 12-h work shift included blood draws (41.2%), dressing wounds (73.5%), caring for IVs, urinary catheters, endotracheal tubes, and/or drains (38.2%), performing a physical examination (55.9%), handling soiled bedpans (50.0%) and soiled linen (51.5%), and performing patient hygiene functions (61.8%).

2.2. qPCR Results

The proportion of potential pathogens detected varied across the three collection visits: *S. aureus* ranged from 41.2% to 52.9%; *Enterococcus* spp. ranged from 52.9% to 61.8%; *C. albicans* ranged from 2.9% to 8.8% and MRSA ranged from 2.9% to 5.9% (Table 1). *S. aureus* and *Enterococcus* spp. co-occurred the most frequently, ranging from 29.4% to 35.3% across collection visits.

**Table 1:** Relative abundances of potential pathogens detected during weekly collection visits on dominant hands of surgical intensive care unit healthcare workers participating in the Healthy Hands Study, July 2011 (N = 34).

Pathogen (targeted gene)	Collection Visit	Ɏ * Mean copies/uL	* Positive (%, n = 34)
*Staphylococcus aureus* (nuc)	1	951.3	41.2
	2	6,623.1	41.2
	3	351.4	52.9
*Enterococcus* spp. (16S)	1	1,702.9	52.9
	2	2,877.1	70.6
	3	1,823.6	61.8
*Candida albicans (18S)*	1	663.8	8.8
	2	336.3	5.9
	3	142.7	2.9
MRSA (mecA|orfX)	1	173.5	2.9
	2	1,405.8	5.9
	3	3,763.5	2.9

Ɏ nuc (~1 copy/cell); *Enterococcus* (~5 16S rRNA copies/cell); *C. albicans* (~100 copies/cell). * qPCR cut-off of 100 copies/ul defined as qPCR limit of detection used to identify a HCW as positive.

### 2.3. Association between Risk Factors for Pathogen Carriage and HCW Hand Microbiota

**Figure 2:**
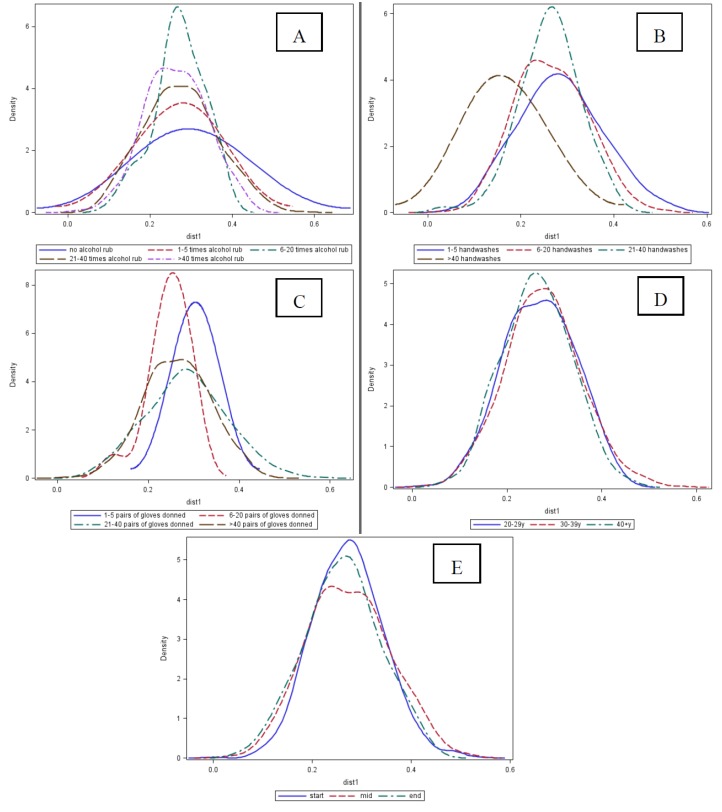
Distributions of weighted UniFrac distance of skin microbiome, by measures of hand hygiene, participant age and time of sample collection. Surgical intensive care unit healthcare workers participating in the Healthy Hands Study, July, 2011 (N = 34 healthcare workers; N = 102 samples). Panel A: Frequency of alcohol rub use (F statistic for means: 1.8, *p*-value = 0.13; F statistic for variances: 8.17, *p*-value < 0.0001); Panel B: Frequency of handwashes (F statistic for means: 4.25, *p*-value: 0.0053; F statistic for variances: 7.27, *p*-value < 0.0001); Panel C: Number of gloves donned (F statistic for means: 7.04, *p*-value = 0.0001; F statistic for variances: 10.05, *p*-value < 0.0001); Panel D: Age (F statistic for means: 1.22, *p*-value = 0.30; F statistic for variances: 2.75, *p*-value = 0.06); and, Panel E: Time within shift (F statistic for means: 2.79, *p*-value = 0.06; F statistic for variances: 1.31, *p*-value = 0.27).

Of all potential risk factors for pathogen carriage, only hand hygiene (*i.e.*, handwashing, alcohol rub use, and donning of gloves) was associated with HCW hand microbiota (Figure 2; see supplemental materials for more detailed analyses). Participants who did not use alcohol rub had a wider distribution of weighted UniFrac distances than all other HCW. However, the mean distance within all levels of alcohol rub use did not differ. All frequencies of handwashing, except those reported as >40 times per 12-h work shift, had similar and notably higher mean distances, indicating higher microbial community diversities. Washing hands >40 times per 12-h work shift had a reduced mean distance of the microbial communities. Donning only 1–5 pairs of gloves per 12-h work shift was associated with a slightly higher mean distance than other frequencies, however, among those who reported donning gloves over 20 times there was a wider distribution range. The distribution of weighted UniFrac distances by age and by time within work shift (e.g., start, middle, or end of work shift) did not differ, suggesting no association between these risk factors for pathogen carriage and the HCW hand microbiota.

### 2.4. Association between HCW Hand Microbiota and Pathogen Carriage

**Figure 3:**
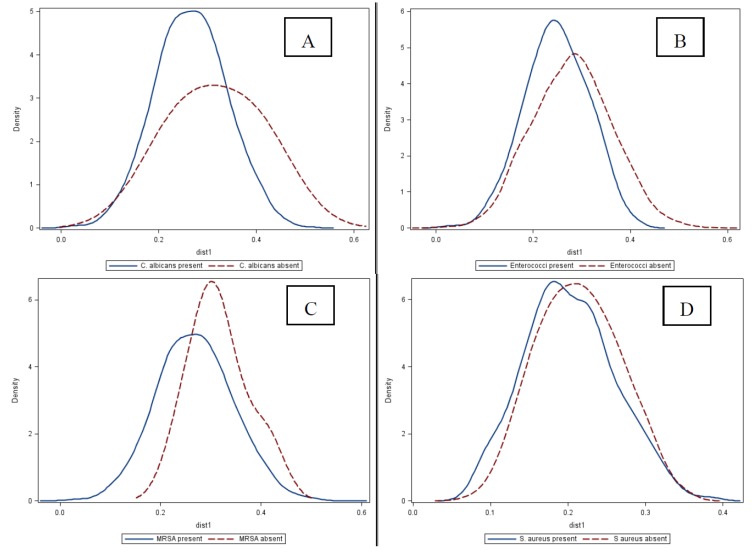
Distributions of weighted UniFrac distance of skin microbiome, by presence and absence of selected potential pathogens. Surgical intensive care unit healthcare workers participating in the Healthy Hands Study, July, 2011 (N = 34 healthcare workers; N = 102 samples). Panel A: *Candida albicans* (t statistics: 2.13, *p*-value = 0.03); Panel B: *Enterococcus* spp. (t statistic: 10.45, *p*-value < 0.0001); Panel C: Methycillin-resistant *Staphylococcus aureus* (MRSA; t statistic: 1.49, *p*-value = 0.13); and, Panel D: *Staphylococcus aureus* (t statistic: 4.81, *p*-value < 0.0001).

In general, the presence of a potential pathogen was associated with a lower mean weighted UniFrac distance (Figure 3): HCW with a lower beta diversity of their hand microbiota were more likely to have a potential pathogen present on their hands than those without.

### 2.5. Discussion

We interrogated the hand microbiome of 34 HCWs at a single intensive care unit to gain insight into the role of skin microbiota in resisting colonization by common nosocomial pathogens, taking into account known behavioral risk factors. Our study is novel in that we uniquely examined the association among hand microbial community structure, potential pathogen carriage, and hand hygiene practices and other factors than modify or mediate the hand microbiota. Analogous to the impact of diet and antibiotic use on the gut microbiota, our results suggest that the hand microbial community structure varies by hand hygiene, and presence of selected potential pathogens.

Overall, both the HCW hand microbiota and other risk factors were associated with the carriage of specific potential pathogens. However, a limitation of this study is the directionality of the association between carriage of potential pathogens and HCW hand microbiota. That is, it may be that the hand microbiota is itself a result of the carriage of certain pathogens or that both the microbiota and the carriage of specific potential pathogens are associated with yet another factor. Despite reports that HCWs performed hand hygiene more than 40 times per 12-h shift, suggesting that the presence of bacteria on their hands were likely colonizers, the distinction between potential pathogen contamination and colonization is not explicitly made in this study. The presence of some microorganisms in the skin microbiota may have an effect on the growth of potential pathogens as well as on the ability of a contaminant to colonize [4,5]. *S. aureus,* once believed to be a “transient colonizer during abnormal conditions”, is now known to be a resident bacterium that somehow turns pathogenic upon disturbance of the individual’s skin microbiota [6,7]. While the consequences of the presence of either a contaminant or a colonizer among a vulnerable population of ICU patients is equally worrisome, proving that the hand microbiota mediates resistance to pathogen colonization rather than contamination is challenging: it is likely that the effect of colonization resistance on the hands is small, and—as we observed—the skin microbial diversity over time highly dynamic.

The prevalence of the selected potential pathogens we found on HCW’s hands is consistent with previous reports. Using qPCR, a Danish longitudinal study of *S. aureus* carriage on the hands of 20 HCW showed that 45% of the participants were positive on all 10 days [8]. We also observed that 45% of HCW were positive over the 3 weeks of our study. Using culture, epidemiologic investigations of HCW hands contaminated with vancomycin-resistant enterococci found 0 to 41% of hands positive [9]. We did not distinguish between resistant and non-resistant enterococci, but found a relatively high prevalence of 62% over 3 weeks. The increased prevalence may reflect the higher sensitivity of qPCR. We detected MRSA in 3.9%, similar to that reported on the fingertips of 523 HCW sampled on 822 occasions, where 38/822 (5%) were positive [10]. The prevalence of *C. albicans* was also similar to that reported in the literature. We found 5.9% of HCW were positive over 3 weeks, compared to 4.4% among 90 swab samples taken from the hands of the nursing staff at a Kuwaiti intensive care unit over an 8 month period [11].

Hand hygiene reduces HAI and microorganisms associated with HAI [12,13]. Given the association between hand microbiota community structure (shown in Figure 3 using UniFrac similarity distances) and carriage of HAI implicated microorganisms, it is possible that hand hygiene modifies the types of pathogens present on the hands through alterations of hand microbiota. Current guidelines on hand hygiene in the health care setting are based on studies that examine transient microorganisms individually rather than by assessing communities of microorganisms (both resident and transient) [12,13]. Studies focusing on hand hygiene product efficacy, for example, might benefit from measuring the hand microbiota (among HCW) in conjunction with targeted indicator species, as demonstrated here. Additional studies focusing on the potential link between hand hygiene, microbiota, and HAI risk among HCWs with more homogeneous hand hygiene practices and pathogen transmission risk, where temporality can be established, are needed to better understand the effect of microbiota diversity on potential pathogen contamination/colonization.

## 3. Experimental Section

### 3.1. Study Population

Healthcare workers were recruited from the University of Michigan (UM) Hospital SICU, a 20-bed critical care unit specializing in patient recovery from major post-operative procedures or those requiring extensive physiological monitoring. To qualify for inclusion, HCW could not have received topical or systemic steroids or antibiotics for a period of 3 months before the start of the study. Physicians were excluded from the study due to their high mobility. The study was presented at staff meetings and the first 35 HCW who met eligibility criteria and gave written consent were included. One HCW was lost to follow-up prior to sample collection leaving a total sample size of 34. The study took place 5–28 July 2011. The study protocol was reviewed and approved by the institutional review board of the UM (IRBMed #HUM00042622).

### 3.2. Survey Instruments for Acquiring Potential Risk Factors for Pathogen Carriage

At enrollment, participants were given a self-administered questionnaire regarding basic demographics, overall health, hand health, hand hygiene practices, and levels of patient contact. Questions were developed based on a literature review that identified elements important in shaping HCW’s microbial community structure [14]. Included was the Hand Skin Assessment, a 7-point (7 = healthiest) self-rating scale used by the participants to assess the current appearance, intactness, moisture content, and sensation of their hands. This scale has been used extensively in previous studies of skin condition, and the scores correlate with other physiologic measures of skin damage [15,16,17,18,19,20].

Upon questionnaire completion, a Visual Scoring of Skin Scale was performed by two trained data collectors (Cronbach's alpha = 0.7; ICC = 0.59, 95%CI: 0.09–0.86), who visually inspected the participant's dominant hand for skin irritation with a 30× magnifying glass. The possible range of scores indicating no observable scale or irritation of any kind to extensive cracking of skin surface, was 0 to 5, respectively; these scores have been highly correlated with participants’ self ratings regarding hand dryness, indicating good validity [17]. Visual assessment is considered a cost-effective, practical and accurate method of evaluating skin irritation [17,21].

We selected our study population from HCW employed at a single ICU, making the assumption that their risk of exposure to the potential pathogens of interest would be similar. In addition, we measured factors that might modify that exposure, such as hand hygiene, time in shift, and types of patient care so we could further adjust for any differences in exposure in the analysis.

### 3.3. Sample Collection

A total of three samples were collected from each HCW at different time points, resulting in a total of 102 samples per collection method (*i.e.*, swab and glove-juice). To minimize sample cross-contamination, study recruiters donned sterile gloves prior to each sample collection. Negative controls consisting only of buffer solution (20 mM Tris pH 8, 2 mM EDTA, and 1.2% Triton X-100) were collected and analyzed for each sampling. The palm, fingertip surfaces, and in-between the fingers of the participant's dominant hand were swabbed using a sterile, cotton-tipped swab soaked in buffer solution. Swab specimens were used for hand microbiota profiling. Immediately after swabbing, the participant's dominant hand was inserted into a sterile, polyethylene bag containing 50 mL buffer solution (0.07 M PBS, 0.1% Tween-80) and massaged through the wall of the bag for 1 minute. This glove-juice sample was then used to detect the presence of potential pathogens of interest. All samples were stored at −20 °C until further processing. HCW were randomly sampled at the start, middle, and end of their shifts; and, were not asked to wash their hands prior to collection, but were also not prevented from doing so. Most HCW performed hand hygiene within 10 min before sample collection, ranging from immediately before to 160 min before (median of 10 min, mean of 24.5 min, std. dev. of 32.3 min). Although investigators did not observe the practices of all participants throughout the study, participants were visited on an unannounced, regular basis by investigators, usually at least once a day, during the data collection period (5–18 July 2011).

### 3.4. DNA Extraction, Purification and Amplification

All swab samples and the pellet of 1 ml of all glove-juice samples were lysed using enzyme cocktail (mutanolysin @ 160U/mL, Rnase A @ 0.07mg/mL, lysostaphin @ 0.16 mg/mL, and lysozyme @ 7mg/mL) for 30 min at 37 °C. The standard protocol for lysing Gram-positive bacterial cell lysates of the PureLink Genomic DNA kit (Invitrogen Corp.: Carlsbad, CA, USA; #K1820-02) was followed for all subsequent steps, with an additional incubation at 95 °C for 2 min, prior to the addition of 96%–100% ethanol to the lysates. Purified genomic DNA were re-suspended in 50 μL of PureLink Genomic Elution Buffer and stored at −80 °C until sent for sequencing.

DNA was tested for PCR competency, using the following procedure. Primers L-V6 (5'-CAACGCGARGAACCTTACC-3') and R-V6 (5'-CAACACGAGCTGACGAC-3') were chosen to amplify the V6 hypervariable region of the 16S rRNA gene [22]. After extraction, 1 uL of the purified genomic DNA was used as template for a 25 uL PCR reaction on a MyCycler Thermal Cycler (Bio-Rad Laboratories, Inc.: Hercules, USA) that included 22.5 uL of Platinum Blue PCR SuperMix (Invitrogen Corp.: Carlsbad, CA, USA; #12580-023) 1 uL of 10 uM primer pair, and 0.5 uL of water. PCR conditions included: 94 °C for 2 min; 30 cycles of (94 °C for 30 s; 55 °C for 30 s; 72 °C for 30 s); and hold at 4 °C. A negative control including all ingredients but with water instead of DNA template was included alongside all test reactions. A constant volume aliquot of each PCR amplification product was run on a 1.5% agarose gel to test PCR competency as well as the approximate amount of product. 10–20 µL of the purified genomic DNA were sent for sequencing at The London Regional Genomics Centre at the University of Western Ontario (London, ON, Canada).

### 3.5. Real-time Quantitative PCR for Pathogen Carriage Detection

Relative abundances of four potential nosocomial pathogens were assessed among all glove-juice samples: *Enterococcus* spp., *Staphylococcus aureus*, methicillin-resistant *Staphylococcus aureus* (MRSA), and *Candida albicans*. These organisms were selected based on the most prevalent ICU pathogens reported to the National Healthcare Safety Network [23]. Primer sets for *Enterococcus* spp., *Staphylococcus aureus*, and MRSA were obtained from the literature, targeting the 16S rRNA gene, the nuc gene, and the single-locus mecA|orfx, respectively (Table 2). The primer set for *Candida albicans* was developed in -house targeting the 18S rRNA gene.

**Table 2:** Primer sets used in the real-time qPCR assays to detect *Enterococcus* spp., *Staphylococcus aureus*, methycillin resistant Staphylococcus aureus (MRSA), and *Candida albicans*.

	Target	Primers (5' → 3')	Reference
*Staphylococcus aureus*	Nuc	GCGATTGATGGTGATACGGTT AGCCAAGCCTTGACGAACTAAAGC	[24]
MRSA	mecA|orfX	TATGATATGCTTCTCC AACGTTTAGGCCCATACACCA	[25]
*Enterococcus* spp.	16S	CCCTTATTGTTAGTTGCCATCATT ACTCGTTGTACTTCCCATTGT	[26]
*Candida albicans*	18S	GGATCGCTTTGACAATGG GCGGGTAGTCCTACCTGATTT	[27]

* *Enterococcus faecalis, E. faecium, E. asini, E. saccharolyticus, E. casseliflavus, E. gallinarum, E. dispar, E. flavescens, E. hirae, E. durans, E. pseudoavium, E. raffinosus, E. avium, E. malodoratus, E. mundtii, E. azikeevi, E. canis, E. gilvus, E. haemoperoxidus, E. hermanniensis, E. moraviensis, E. pallens, E. phoeniculicola, E. villorum, E. rottae.*

We optimized real-time, quantitative PCR (qPCR) protocols using SYBR Green technology (SsoFast EvaGreen Supermix (Bio-Rad; #172-5200)) on a CFX-96 thermocycler platform (Bio-Rad; #185-5195). Final optimal conditions are: 98 °C for 2 min; 40 cycles of (98 °C for 1 s; 60 °C for 1 s—*Enterococci* spp.) (98 °C for 4 s; 60 °C for 4 s—*S. aureus*) (98 °C for 2 s; 56 °C for 2 s—MRSA) (98 °C for 1 s; 63 °C for 1 s—*C. albicans*); and 65–95 °C (increment of 0.5 °C) for 5 s. Standard curves from a 10-fold dilution series (10^8^–10^2^) were run using genomic DNA or cloned target DNA from the following positive controls, obtained from the UM Clinical Microbiology and Virology Laboratories and the Molecular and Clinical Epidemiology Laboratory: *Enterococcus* spp. (ATCC# 29212), *S. aureus* (ATCC# 25923), MRSA (ATCC# 1026), and *C. albicans* (ATCC# MYA-2876).

### 3.6. Statistics

Four separate marginal models that accounted for repeated measures were fit to assess the association between potential risk factors and pathogen carriage (Supplementary Materials). Potential risk factors included frequency of handwashing, alcohol rub, and gloves, age, and time within work shift (start, middle, and end). This type of model describes the fixed effects of covariates on the population mean response over the study period time. A backward selection model fitting strategy—where all covariates of interest were included and removed one at a time if non-significant—was done to obtain mean predicted values of potential pathogen carriage among the HCW SICU population. The outcome variable, that is, the level of pathogen as measured by qPCR, was log transformed to fit a normal distribution. This also facilitated interpretation, since one unit increase in copy number is not biologically relevant. Variance-covariance matrices of the random errors were compared based on their model fit criteria. The most parsimonious R structure that yielded the lowest AIC and BIC, was selected. Most fixed effects that were non-significant, based on the Type 3 Tests, were removed from the model. Residual diagnostics were then evaluated (Figure S1) [28].

The UniFrac distance metric measures the difference between two groups in terms of the phylogenetic branch length unique to one group or the other. The branch lengths are proportional to the number of base changes in the V6 16S rRNA gene. The weighted UniFrac, which weights branches based on relative abundances, was used. Associations between the HCW hand microbiota and carriage of potential pathogens (Figure 1), were assessed by comparing the distribution of the weighted UniFrac distances among HCW with *Enterococcus* spp. or *S. aureus* present on their hands with those without. Similarly, to investigate the association between the significant risk factors for pathogen carriage and the HCW hand microbiota (Figure 1), the distribution of weighted UniFrac distances between groups of HCW that belonged to a certain category of a potential risk factor, were examined. A higher mean weighted UniFrac distance reflects a group of individuals with a more diverse microbial community. A wider distance distribution reflects a group of individuals that comprise highly diverse and less diverse microbial communities. Since HCW sampled using swabs were statistically no more similar to themselves over the 3 time points than to other HCW in the study, weighted UniFrac distances were obtained from samples collected via swabs analyzed collectively [29].

## 4. Conclusions

In summary, our primary finding was that after correcting for individual risk of exposure to the selected potential pathogens, differences in carriage of these potential pathogens might be attributed to the structure of the hand microbial community. Previous studies have demonstrated that commensal microbiota can resist pathogen invasion or, if disrupted, be neutral to or even enhance pathogen invasion [30,31]. Our results are consistent with these previous studies, and suggest that this is true even for the hands of HCW that are washed up to 40 times a day. Additionally, we found that HCW's age, hand hygiene, and work shift were significant risk factors for carriage of potential pathogens, as detected by qPCR. We also found an association between these risk factors and their overall hand microbiota structure, as profiled using 16S rRNA sequencing. Interestingly, their overall microbiota structure was also associated with potential pathogen carriage, suggesting the possibility of its mediating or modifying role in the relationship between age, hand hygiene, work shift, and pathogen carriage. Moreover, hand microbiota community structure may act as a biomarker of pathogen carriage, and modifying the structure may provide an additional strategy for limiting pathogen carriage among HCW. Further studies in larger populations and more diverse clinical settings are needed to better elucidate the potential protective or detrimental roles that hand microbiota community structure plays in the transmission of hospital-acquired infections.

## Supplementary Files

Supplementary File 1 Supplemental Materials (PDF, 339 KB)
