# Supplementary material for: Healthcare Workers’ Hand Microbiome May Mediate Carriage of Hospital Pathogens
Source: Pathogens. 2013 Dec 27;3(1):1–13. doi: 10.3390/pathogens3010001 (PMC4235731; doi:10.3390/pathogens3010001)

Supplemental Materials

Table S1. Analysis of mixed model parameter estimates, accounting for repeated measures, predicting mean level of *Staphylococcus aureus* and *Enterococcus* spp., as measured by the log of gene copy numbers detected using qPCR.

	Staphylococcus aureus			***Enterococcus* spp.**		
**Parameter**	**Estimate**	**0.95 CL**	***p*-value**	**Estimate**	**0.95 CL**	***p*-value**
Intercept	3.063	(1.827, 4.299)	<0.0001	2.779	(1.197, 4.360)	0.0012
Age				0.087	(0.0543, 0.121)	<0.0001
Handwashes						
1–5 times	REF	REF	REF	REF	REF	REF
6–20 times	−0.045	(−0.626, 0.535)	0.87	0.462	(−0.513, 1.437)	0.34
21–40 times	−0.354	(−1.172, 0.464)	0.38	−0.493	(−1.555, 0.570)	0.35
>40 times	−0.125	(−0.847, 0.597)	0.72	−2.382	(−4.318, -0.446)	0.02
Alcohol Rub Use						
None	REF	REF	REF			
1−5 times	−1.546	(−2.892, −0.201)	0.03			
6−20 times	0.933	(0.207, 1.658)	0.01			
21−40 times	−0.017	(−0.713, 0.679)	0.96			
>40 times	0.115	(−0.694, 0.924)	0.77			
Gloves Donned						
1−5 times	REF	REF	REF			
6−20 times	1.795	(0.272, 3.318)	0.02			
21−40 times	2.812	(1.592, 4.032)	0.0001			
>40 times	2.709	(1.384, 4.034)	0.0004			
Time within Shift						
Start	REF	REF	REF			
Mid	–0.833	(–1.443, –0.222)	0.01			
End	–0.366	(–1.279, 0.548)	0.41			

Note: REF = referent group; CL = confidence limits (p-value based on 95% level of significance).

In comparison to not using any alcohol rub, HCW who used it 1–5 times per work shift had a 20% decreased level of *S. aureus* (*p*-value = 0.03). Higher frequency of glove use during the 12-h work shift was associated with a greater frequency of *S. aureus* on hands. Sampling HCW in the middle of their work shift was associated with a 43% lower amount of *S. aureus* in comparison to the start of their shift (*p*-value = 0.01). Type 3 tests of fixed effects show that frequency of handwashes (F = 4.95, *p*-value = 0.007) and age (F = 29.24, *p*-value < 0.0001) were statistically significantly associated with

frequency of *Enterococcus* spp. carriage. For each year increase in age, there was an approximate 9% greater frequency of *Enterococcus* spp. carriage (p-value < 0.0001). Washing hands with soap and water > 40 times per 12-h work shift resulted in a 9% lower frequency of *Enterococcus* spp. carriage (p-value = 0.02). The Toeplitz R structure was selected for estimating the variances and covariances of the random errors since it assumes no exponential decay. Models predicting *C. albicans* carriage and MRSA carriage were not fit due to low prevalence of these microbes. Type 3 tests of fixed effects show that frequency of alcohol rub use (F = 7.86, p-value = 0.0006), frequency of glove use (F = 8.59, p-value = 0.0007), and time within shift (e.g. start, middle or end of work shift) (F = 4.28, p-value = 0.03), were statistically significantly associated with frequency of *S. aureus* carriage.

In examining the association between hand hygiene practices and other factors that may mediate or modify the hand microbiota, we found that the frequency of alcohol rub use and glove use, and sampling time during the work shift were significantly associated with *S. aureus* carriage. In comparison to not using any alcohol rub, HCW who used rub 1–5 times/work shift had a 20% decrease in the amount of *S. aureus*. Sampling HCW hands in the middle of their work shift was associated with a 43% lower amount of *S. aureus*, in comparison to the start of their shift, which may reflect the cumulative effect of hand hygiene performed throughout their 12-h shifts. Unlike *S. aureus* carriage, *Enterococcus* spp. carriage was statistically significantly associated with the frequency of handwashing. Curiously, the use of alcohol rub was not associated with the frequency of *Enterococcus* spp. carriage, possibly reflecting a variable effect of alcohol on different bacterial species. For every year increase in age, there was an approximate 9% greater frequency of *Enterococcus* spp. carriage. This could be explained by either the modifying effects of their hand microbiota, the activities performed outside the SICU, or simply the physiologic and/or anatomic changes in the skin associated with aging. Additionally, it is possible that older HCW care for patients with a higher burden of *Enterococcus* spp.

Table S2. Model Fitting Criteria.

Model	Covariance Structure	Number of Covariance Parameters	AIC	BIC
S. aureus	Unstructured	6	1415.4	1424.5
	Compound Symmetry	2	1442.9	1446.0
	Compound Symmetry Heterogeneous*	4	1412.6	1421.0
	**Toeplitz**	**3**	**1440.7**	**1445.3**
	Toeplitz Heterogeneous*	5	1413.4	1421.0
	Autoregressive*	4	1413.0	1419.1
Enterococcus spp.	Unstructured	6	1643.6	1652.8
	Compound Symmetry	2	1701.9	1704.9
	Compound Symmetry Heterogeneous*	4	1643.5	1649.6
	**Toeplitz**	**3**	**1692.4**	**1697.0**
	Toeplitz Heterogeneous**	5	-	-
	Autoregressive*	4	1640.1	1646.2

*Convergence criteria met but final hessian is not positive definite.

**Convergence criteria not met.

The models with the Toeplitz R structure were selected as the final models. Among those that had the convergence criteria fully met, they were the most parsimonious (least parameterized), with the lowest AIC and BIC results. The final models were: log (*S. aureus*$_i$) = (HANDWASH)$_i\beta$ + (ALCOHOL RUB)$_i\beta$ + (GLOVE USE)$_i\beta$ + (SHIFT)$_i\beta$ + ε_i; and, log (*Enterococcus* spp.$_i$) = (HANDWASH)$_i\beta$ + (AGE)$_i\beta$ + ε_i, where, $\varepsilon_i \sim N(0,R_i)$.

Table S3. Type 3 Tests of Fixed Effects.

Model	Effect	Numerator DF	Denominator DF	*F*-value	*p*-value
S. aureus	SOAPWK	3	20	0.48	0.6982
	ALCWK	4	20	7.86	0.0006
	GLOVEWK	3	20	8.59	0.0007
	SHIFT	2	15	4.28	0.0339
Enterococcus spp.	SOAPWK	3	28	4.95	0.0070
	AGE	1	28	29.24	<0.0001

The type 3 tests of fixed effects show the statistical significance of each variable given all others listed in the model.

According to the plots, the variability of the standardized residuals appears similar across all predicted values (*i.e.*, homogeneous). Also, the distribution of these residuals is normal (with very few outliers at both ends).

Figure S1. *S. aureus* (Panel A) and *Enterococcus* spp. (Panel B) Model Diagnostics.

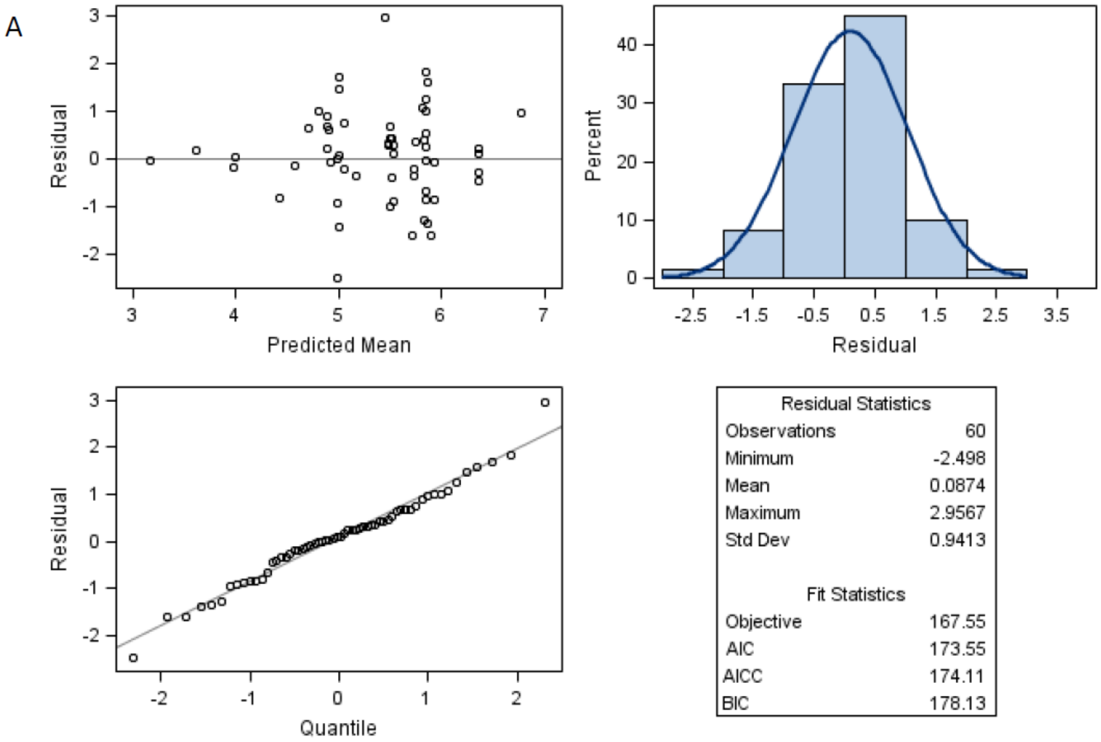

Figure SI. *Cont.*

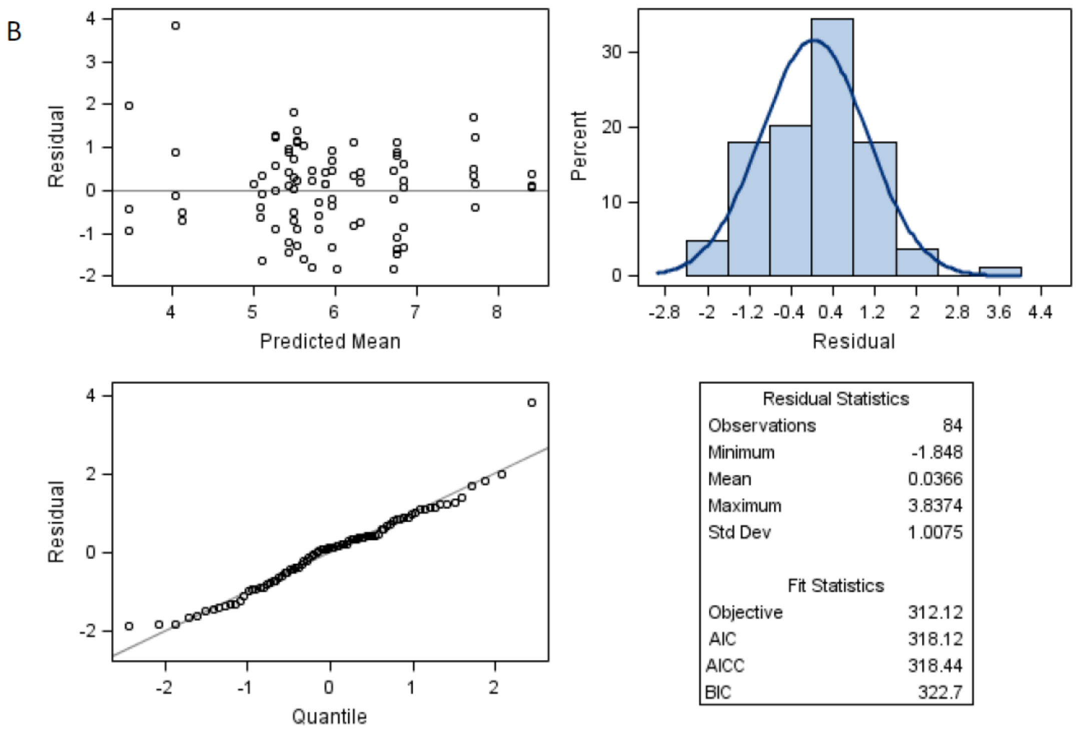

Supplement: Supplementary File 1 — Supplemental Materials (PDF, 339 KB) [file pathogens-03-00001-s001.pdf]
